# Safety from Exploding Vulcanizers

**Published:** 1870-03

**Authors:** W. E. Driscoll

**Affiliations:** Bedford, Ind.


### SAFETY FROM EXPLODING VULCANIZERS.

BY W. E. DRISCOLL, BEDFORD, IND.

Now that the attention of all is directed to the danger to which we are subjected from vulcanizers exploding, and that, too, when they get old and weak, in spite of all the thermometers, fusible alloys, alarm bells and safety-valves that have been devised, something that can be relied upon as safe will stand a good chance of general adoption.

I ordered, at the wagon-maker's, an oaken box, two feet square by one foot deep, of lumber three inches thick, to be bound together by ten iron rods, one-inch size, running entirely through, from side to side, and inserted through flat iron bars, of suitable size. The legs, also, are fastened by the ends of four of these rods, and elevate the "safe" to a level with the breast of the operator. The feet are fastened firmly to the floor. One-third of the top is enclosed on front side—rest open. Bottom so as to be easily blown out. A small hole through front side gives a view of the flame, which is regulated by reaching in at the top.

With this apparatus, I propose to use my vulcanizer—now seven years old—until it *does burst*.

Who will suggest something better?

A far safer method than this is to adopt Dr. Palmer's method. See page 67, Feb. No. of REGISTER.—ED.

